# Properties of Degradable Polyhydroxyalkanoates (PHAs) Synthesized by a New Strain, *Cupriavidus necator* IBP/SFU-1, from Various Carbon Sources

**DOI:** 10.3390/polym13183142

**Published:** 2021-09-17

**Authors:** Natalia O. Zhila, Kristina Yu. Sapozhnikova, Evgeniy G. Kiselev, Alexander D. Vasiliev, Ivan V. Nemtsev, Ekaterina I. Shishatskaya, Tatiana G. Volova

**Affiliations:** 1 Basic Department of Biotechnology, School of Fundamental Biology and Biotechnology, Siberian Federal University, 79 Svobodnyi Av., 660041 Krasnoyarsk, Russia; kristina.sap@list.ru (K.Y.S.); evgeniygek@gmail.com (E.G.K.); adva@iph.krasn.ru (A.D.V.); ivan_nemtsev@mail.ru (I.V.N.); shishatskaya@inbox.ru (E.I.S.); volova45@mail.ru (T.G.V.); 2 Federal Research Center “Krasnoyarsk Science Center SB RAS”, Institute of Biophysics SB RAS, 50/50 Akademgorodok, 660036 Krasnoyarsk, Russia; 3 Federal Research Center “Krasnoyarsk Science Center SB RAS”, L.V. Kirensky Institute of Physics SB RAS, 50/38 Akademgorodok, 660036 Krasnoyarsk, Russia; 4 Federal Research Center “Krasnoyarsk Science Center of the Siberian Branch of the Russian Academy of Sciences”, 50 Akademgorodok, 660036 Krasnoyarsk, Russia

**Keywords:** *Cupriavidus necator* IBP/SFU-1, cell growth and PHA synthesis, various carbon sources, PHA composition and properties, polymer films

## Abstract

The bacterial strain isolated from soil was identified as *Cupriavidus necator* IBP/SFU-1 and investigated as a PHA producer. The strain was found to be able to grow and synthesize PHAs under autotrophic conditions and showed a broad organotrophic potential towards different carbon sources: sugars, glycerol, fatty acids, and plant oils. The highest cell concentrations (7–8 g/L) and PHA contents were produced from oleic acid (78%), fructose, glucose, and palm oil (over 80%). The type of the carbon source influenced the PHA chemical composition and properties: when grown on oleic acid, the strain synthesized the P(3HB-co-3HV) copolymer; on plant oils, the P(3HB-co-3HV-co-3HHx) terpolymer, and on the other substrates, the P(3HB) homopolymer. The type of the carbon source influenced molecular-weight properties of PHAs: P(3HB) synthesized under autotrophic growth conditions, from CO_2_, had the highest number-average (290 ± 15 kDa) and weight-average (850 ± 25 kDa) molecular weights and the lowest polydispersity (2.9 ± 0.2); polymers synthesized from organic carbon sources showed increased polydispersity and reduced molecular weight. The carbon source was not found to affect the degree of crystallinity and thermal properties of the PHAs. The type of the carbon source determined not only PHA composition and molecular weight but also surface microstructure and porosity of the polymer films. The new strain can be recommended as a promising P(3HB) producer from palm oil, oleic acid, and sugars (fructose and glucose) and as a producer of P(3HB-co-3HV) from oleic acid and P(3HB-co-3HV-co-3HHx) from palm oil.

## 1. Introduction

Degradable polyhydroxyalkanoates (PHAs) are regarded as the real candidate to gradually replace non-degradable synthetic polymers, which are now used extensively, posing a global environmental problem [1,2,3,4,5,6]. Polymers of hydroxy-derived alkanoic acids, polyhydroxyalkanoates (PHAs), comprise polymers with various chemical compositions, which have valuable properties including biocompatibility and biodegradability. Therefore, these biopolymers are promising materials of the 21st century for diverse applications from municipal engineering and agriculture to pharmacology and biomedicine [7,8,9,10,11,12].

Polyhydroxyalkanoates are energy and carbon storage macromolecules of a cell, which are synthesized by prokaryotes under specific conditions of unbalanced growth when synthesis of the main compounds (protein and nucleic acids) is limited, but the medium contains excess carbon [13,14]. Conditions under which the direction of cell anabolism changes from protein synthesis to PHA synthesis and accumulation are determined by the redox state of cytoplasm and intracellular concentrations of pyruvate and available CoA [15]. During balanced growth of PHA-producing bacteria, pyruvate and reducing equivalents (NADH and NADPH) are mainly expended in the tricarboxylic acid cycle to form amino acids and to transform energy in the cell. The level of available CoA remains high, which hinders polymer synthesis. Under unbalanced growth, in a nitrogen-free or nutrient-deficient medium, pyruvate does not enter the tricarboxylic acid cycle but is carboxylated, forming acetyl-CoA. The level of available CoA is low, which is a favorable condition for activation of the enzymes involved in the intracellular cycle of storage macromolecules such as PHAs.

PHA production is a purely biological intracellular synthesis performed by bacterial cells grown on various carbon sources. PHAs are represented by polymers with different chemical structures and considerably diverse properties: molecular-weight and temperature characteristics, degree of crystallinity, and biodegradation behavior and rates [6,16,17,18,19,20,21]. PHAs may be potentially produced from substrates that can be reduced to different degrees and that vary in energy content and cost such as individual compounds and complex substrates, including industrial wastes [22]. Over 300 microorganisms are known to produce and accumulate PHAs, they are represented by both wild-type and genetically modified strains.

The hydrogen-oxidizing bacteria of the *Cupriavidus* genus (formerly *Wautersia*, *Ralstonia*, *Alcaligenes*, *Hydrogenomonas*) are promising PHA producers [23]. Being autotrophic, they are able to synthesize PHAs from mixtures of carbon dioxide and hydrogen [24,25,26,27,28]; moreover, they have a broad organotrophic potential and are capable of synthesizing PHAs with various chemical structures using diverse substrates [9,11,29,30,31]. Because of the strong intracellular system for PHA synthesis and special growth physiology, *Cupriavidus* strains synthesize very high PHA concentrations. Even on a complete nutrient medium, in the middle of the linear growth phase, these microorganisms stop synthesizing protein and begin producing poly-3-hydroxybutyrate (the first and best-studied PHA) [24,25]. However, the challenges of using most of the *Cupriavidus* species include their narrow trophic potential towards sugars and ability to assimilate only fructose for their growth. Another issue is that they are capable of synthesizing only short-chain-length PHAs—the P(3HB) homopolymer and copolymers of 3HB and valerate, P(3HB-co-3HV). A usual way to overcome these limitations is to produce mutant organisms. Therefore, the isolation of highly productive wild-type strains, which have broader organotrophic potential and are able to synthesize PHA copolymers composed of short- and medium-chain-length monomers, can considerably enhance the potential of this genus. Despite the pressing need for degradable polymeric materials and the high attractiveness of PHAs, their high cost and the complications associated with technical and technological processes limit their manufacturing scale and reduce their applications [32,33]. The major challenge of PHA biotechnology is optimizing the entire biotechnology of PHA synthesis, primarily, by using new productive strains, which are capable of growing not only on sugars but also on various available carbon sources, including inexpensive plant oils, glycerol (which is a by-product of large-scale biodiesel production), etc., and are able to synthesize PHA copolymers with different chemical compositions, containing short- and medium-chain-length monomers and showing better performance.

The present study investigated the properties of PHAs synthesized by the new strain, *Cupriavidus necator* IBP/SFU-1, from various carbon sources.

## 2. Materials and Methods

### 2.1. Isolation of the Strain

The strain is maintained in the Collection of Chemolithotrophic Cultures of the Laboratory of Chemoautotrophic Biosynthesis at the Institute of Biophysics SB RAS. The strain was isolated from soil (Krasnoyarsk, Siberia, Russia).

### 2.2. Identification of the Bacterial Strain

The bacterial strain was identified using conventional methods based on the culture and morphological characters, standard biochemical tests [34,35], sequencing of a region of the 16S rRNA gene, and the Matrix-Assisted Laser Desorption/Ionization Time-of-Flight (MALDI-TOF) mass spectrometry method, performed using MALDI-TOF MS Microflex (Bruker Daltonics, Bremen, Germany), based on the automatic analysis of mass spectra of protein molecules specific for the microorganism species.

Phylogenetic analysis of the strain was performed using the Neighbor-Joining method [36]. The evolutionary distances were computed using the Maximum Composite Likelihood method [37] and were in the units of the number of base substitutions per site. Codon positions included were 1st + 2nd + 3rd + Noncoding. All ambiguous positions were removed for each sequence pair (pairwise deletion option). Phylogenetic and molecular evolutionary analyses were conducted using the MEGA software package version X [38].

### 2.3. Cultivation of Cupriavidus Necator IBP/SFU-1 Cells

The aseptic cultivation of bacterial cells was conducted under autotrophic growth conditions in Schlegel’s mineral medium, on the CO_2_:O_2_:H_2_ gas mixture (1:2:7 *v*/*v*%). Under heterotrophic growth conditions, the medium contained one of the following carbon and energy sources at a concentration of 10–15 g/L: fructose (Panreac, Barcelona, Spain) and glucose (Khimreactivsnab, Ufa, Russia); purified glycerol (Corporate Oleon, Emmerich, Germany); plant oils including refined sunflower seed oil and Siberian oilseed oil (“Zolotaya semechka”, Rostov-on-Don, Russia) and bleached refined deodorized palm oil (“Oil de Luxe”, Surabaya, Indonesia); fatty acids including palmitic (Vekton, Russia), myristic (Merck, Bandar Sunway, Malaysia), lauric (Merck, Malaysia), and oleic (Ekos-1, Moscow, Russia) acids.

Fermentation was conducted in 0.5-L flasks containing 200 mL of the medium in an Innova 44 constant temperature incubator shaker (“New Brunswick Scientific”, Edison, NJ, USA) at 200 rpm and 30 °C. The cell cultivation process was described in detail elsewhere [39]; production parameters of the culture such as cell concentration (X, g/L), intracellular polymer content (g/L and % of CDW), and yield coefficients from the carbon source (Y, g/g) were determined using conventional methods.

Biomass fermentation productivity was determined using the formula:

P_x_ = (X_n_ − X_0_)/T,

where X_0_, X_n_ are concentrations of biomass at the start and end of fermentation, respectively, g/L, T is fermentation duration h^−1^.

The yield coefficients of the biomass and polymer, Y_x_ and Y_p_, from the substrates were calculated using the following formula:

Y_x_ = ΔX/∆S, Y_p_ = ΔPHA/∆S, (1)

where ΔX and ΔP are the difference between the initial and final biomass and polymer content, g, and ∆S is the consumed substrate, g.

### 2.4. Analytical Procedures

The intracellular content and composition of PHAs were determined by chromatography of the preliminarily derived methyl ester fatty acids using gas chromatography-mass spectrometry (GC-MS) (7890/5975C, Agilent Technologies, Santa Clara, CA, USA) [40].

The lipids were extracted from wet biomass with the chloroform–methanol mixture (2:1, *v*/*v*). Cells grown on the medium with plant oils were subjected to preliminary treatment with hexane to remove the substrates. In the resulting extract, PHAs were separated from the lipids by precipitation with a double volume of hexane. The lipid extract was dried, and after methyl esterification with H_2_SO_4_:methanol (1:20) solution that lasted 2 h at 80 °C, fatty acid methyl esters (FAMEs) were analyzed using GC-MS (7890/5975C, Agilent Technologies, USA).

### 2.5. PHA Properties

The physicochemical properties of high-purity PHA specimens were determined using modern physicochemical methods, which have been described in detail elsewhere [41,42]. Molecular-weight properties of the polymers (weight-average (M_w_) and number-average (M_n_) molecular weights and polydispersity (Ð)) were measured. Melting point (T_melt_) and thermal degradation temperature (temperature at which sample mass loss begins, T_degr_) were measured using a DSC-1 differential scanning calorimeter (Mettler Toledo, Schwerzenbac, Switzerland) and TGA (Mettler Toledo, Schwerzenbac, Switzerland), respectively. A 3–5 mg sample was heated to 200 °C at a rate of 5 °C/min; the sample was held at 200°C for 1 min, cooled to −20 °C at a rate of 5 °C/min and held for 4 min. Then, the sample was reheated at a rate of 5 °C/min (DSC). A 3–5 mg sample was heated to 450 °C at a rate of 10 °C/min (TGA). Thermograms were analyzed using the “StarE”software. X-ray examination was performed using a D8ADVANCE powder diffractometer (Bruker AXS, Karlsruhe, Germany) equipped with a VANTEC linear detector; the degree of crystallinity (C_x_) was calculated as a ratio of the total area of crystalline peaks to the total area of the radiogram (the crystalline + amorphous components

### 2.6. Preparation and Investigation of Polymer Films

Films were prepared by casting a 2% polymer solution in dichloromethane in degreased Teflon-coated molds, and then the films were left to stay in a laminar flow cabinet (Labconco, Kansas City, MO, USA) for 72 h at room temperature. The surface microstructure of PHA films was analyzed using scanning electron microscopy (FE-SEM S 5500 high-resolution scanning electron microscope Hitachi, Tokyo, Japan). Prior to microscopy, the samples were sputter-coated with platinum (at 25 mA, for 60 s), using an EM ACE200 (“Leica”, Wetzlar, Germany). Water contact angles of the films were studied with a Drop Shape Analyzer-DSA-25E (Krüss, Hamburg, Germany) using the DSA-4 software for Windows. Porosity of the films was determined manually from SEM images using a software package for digital image analysis (free open-source software package for scientific analysis, editing, and processing of raster images), ImageJ v1.53d.

### 2.7. Statistics

Statistical analysis of the results was performed using the standard software package of Microsoft Excel. Arithmetic means and standard deviations were found.

## 3. Results and Discussion

### 3.1. Characterization of the Strain Cupriavidus Necator IBP/SFU-1

The following morpho-cultural characteristics of *Cupriavidus necator* IBP/SFU-1 were determined. The cells were motile 0.3–0.5 × 1.2–2.0-µm rods, which formed cream-colored, smooth colonies on peptone agar. The colonies had the obvious single-center morphology and the colony edge was smooth. The diameter of the 7-day colonies was 3–5 mm, with their number per Petri dish no more than 10.

Biochemically, *Cupriavidus necator* IBP/SFU-1 is an obligate aerobic organism and a facultative chemolitho-organotroph; the strain is capable of growing on CO_2_ and H_2_ as sole carbon and energy sources, respectively. As a nitrogen source, the strain utilizes nitrates, ammonium salts, urea, and amino acids. It is oxidase positive. The strain does not have hydrolytic enzymes, does not liquefy gelatin, and does not hydrolyze starch. Of the tested sugars, the strain utilizes fructose and glucose. It is capable of growing on the media with organic acids and amino acids. It does not need growth factors; it is an antibiotic-resistant strain. The optimal growth temperature is 30–33 °C, pH 7.0 ± 0.2.

Identification of the composition of lipid fatty acids, which is a taxonomic characteristic, shows that the FAs of the strain are represented by the fatty acids containing 12 to 19 carbons, palmitic acid being the predominantly saturated acid (46.2%). Palmitoleic (16:1ω7) and cis-vaccenic acids are the main monoenoic acids, constituting 18.3–27.6 and 22.9–24.2%, respectively. The saturated/unsaturated FA ratio is 0.9 under autotrophic conditions and 1.4 on fructose (data not shown).

In *Cupriavidus necator* IBP/SFU-1, the nucleotide sequence of the 16S rNA gene contained 1482 bp. The obtained nucleotide sequence of the 16S rNA gene was deposited in the GenBank database (Accession Number MW680848.1).

**Figure 1:**
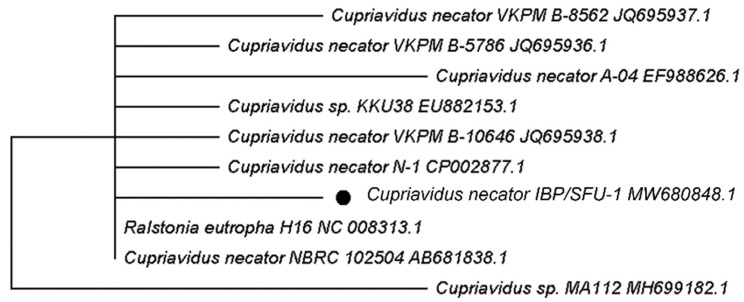
Neighbor-joining phylogenetic tree of *Cupriadivus necator* IBP/SFU-1 and related bacteria based on 16S rRNA sequence comparisons. Accession numbers are given. Bar, 0.0005 substitutions per nucleotide position.

The phylogenetic position of strain IBP/SFU-1 within the genus *Cupriavidus* is shown in Figure 1. Phylogenetic analysis showed that the strain IBP/SFU-1 represented a member of the genus *Cupriavidus.* Comparative 16S rRNA gene sequence analysis revealed that the strain IBP/SFU-1 had a high degree of similarity with other species of the genus *Cupriavidus*.

### 3.2. Cupriavidus Necator IBP/SFU-1 Growth and PHA Synthesis on Various Carbon Sources

The study showed that the strain C. necator IBP/SFU-1 was capable of growing and synthesizing PHAs on various carbon sources: CO_2_, sugars, glycerol, fatty acids, and plant oils. Hence, there are different PHA synthesis pathways in this strain. Eight possible biochemical pathways of PHA synthesis were described in the review by Professor Chen [16]. The first pathway, involving three key enzymes, β-ketothiolase, NADPH-dependent acetoacetyl-CoA reductase, and PHA synthase, occurs in hydrogen-oxidizing bacteria including *Cupriavidus necator*. This pathway was also found in Aeromonas hydrophila, Pseudomonas stutzeri, and Pseudomonas oleovorans [16].

**Figure 2:**
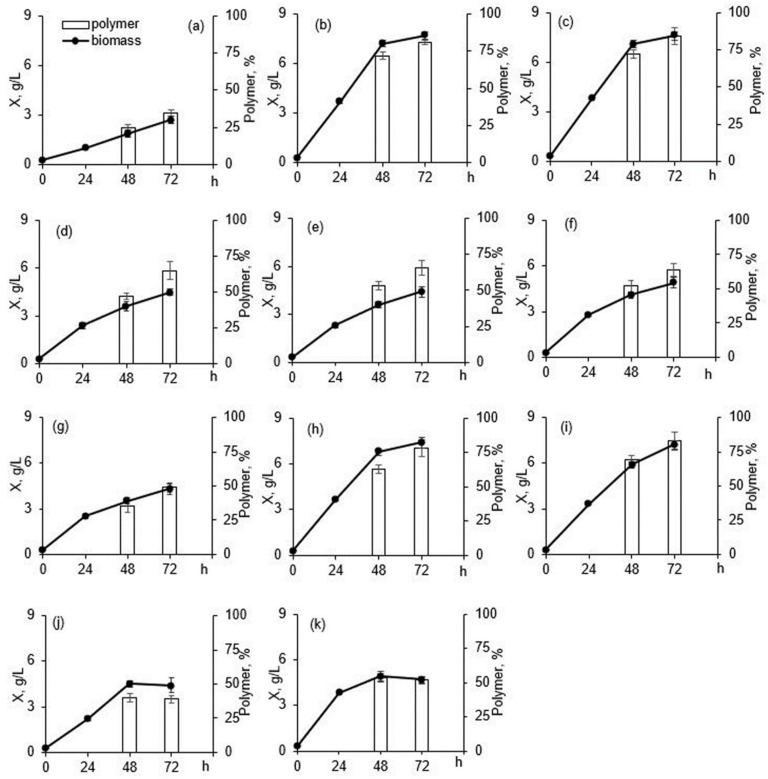
Cell dry weight and PHA production by *Cupriavidus necator* IBP/SFU-1 grown on different substrates. (**a**) CO_2_, (**b**) fructose, (**c**) glucose, (**d**) glycerol, (**e**) lauric acid, (**f**) myristic acid, (**g**) palmitic acid, (**h**) oleic acid, (**i**) palm oil, (**j**) sunflower seed oil, (**k**) Siberian oilseed oil.

Production parameters of the batch culture of C. necator IBP/SFU-1 grown on different carbon sources are shown in Figure 2. The strain was found to be able to synthesize PHAs in autotrophic suspension culture with CO_2_ as the sole carbon source and H_2_ oxidation reaction as the energy source. Cell biomass concentration and polymer content in the three-day culture were 2.6 g/L and 35.1%, respectively. That was comparable to the corresponding parameters achieved using *Cupriavidus necator* Z-1 [25] but inferior to the parameters of the strains maintained in the collection of the Institute of Biophysics SB RAS, *Cupriavidus necator* B5786 and *Cupriavidus necator* B-10646, which, when grown under the same conditions, produced 3.5–4.0 g/L of cell biomass and 60–70% (or even higher) polymer content [28,43]. However, C. necator IBP/SFU-1 is a newly isolated strain, which has been mainly maintained in the stock culture on agar medium. In contrast to *Cupriavidus necator* B 5786 and C. eutrophus B-10646, it has not been used in suspension culture for long periods, has not been selected based on its growth rate, and has not been adapted to the mode of PHA accumulation on CO_2_. Therefore, further research may lead to the enhancement of production parameters of C. necator IBP/SFU-1.

The new strain, C. necator IBP/SFU-1, has a broad organotrophic potential and is able to synthesize high PHA concentrations from various organic substrates such as sugars, glycerol, fatty acids (FAs), and plant oils (Figure 2).

Sugars are the most common substrate for a variety of microorganisms, including PHA producers, which are able to metabolize hexoses for growth. Although the only sugar utilized by hydrogen-oxidizing bacteria is fructose, they readily undergo mutations and acquire the ability to metabolize glucose. The carbohydrate that is generally used to reach high PHA concentrations is ketohexose (fructose), which is phosphorylated by fructokinase into fructose-6-phosphate, which is then isomerized to glucose-6-phosphate, transformed to phosphogluconate, and metabolized via the Entner–Doudoroff pathway. When bacterial cells are grown on glucose instead of fructose, glucose is metabolized via the Entner–Doudoroff pathway to form pyruvate, which may be transformed by dehydrogenase into acetyl-CoA, the central intermediate component in reactions of PHA synthesis [44].

The strain C. necator IBP/SFU-1 was found to be capable of metabolizing glucose, and there was no need to go through a lengthy process of isolation on the selective medium with glucose as the sole carbon source, in contrast to the production of the glucose-utilizing mutant strain *Cupriavidus necator* B 8562 from the parent strain *Cupriavidus necator* B 5786 [45]. Cultivation of C. necator IBP/SFU-1 on fructose and glucose resulted in similar cell concentrations and PHA contents: cell concentrations were approximately 7.0 and 7.7 g/L (productivity of 2.33 and 2.57 g/L d, respectively), and PHA contents were 81.1 (5.68 g/L) and 84.3% (6.33 g/L). These values were slightly lower than the results achieved in the 72-h cultivation of the highly productive strain C. necator B-10646, whose cell concentration reached 8.4 g/L and PHA content 92% [43], and similar to, or higher, than the results reported by other authors for such strains as *Cupriavidus necator* H16 [46,47], *Cupriavidus necator* NCIMB 11599 [48], *Cupriavidus necator* B 8562 [45], and *Cupriavidus necator* A-04 [30]. The new strain consumed similar amounts of fructose and glucose, 2.8 ± 0.2 g/g cell biomass and 3.3 ± 0.3 g/g polymer, and yield coefficients were Y_x_ 0.36 ± 0.03 and Y_PHA_ 0.30 ± 0.03.

A promising source for PHA production by various bacteria, including Cupriavidus, is glycerol, a byproduct of the large-scale and steadily growing biodiesel production [49]. In contrast to glucose, which is metabolized via the Entner–Doudoroff pathway to form pyruvate, which is then transformed by dehydrogenase into acetyl-CoA, glycerol can also be metabolized to form pyruvate but through the intermediate compound, glyceraldehyde-3-phosphate [50].

The ability of the strain *C. necator* IBP/SFU-1 to synthesize PHA from glycerol was investigated in experiments with purified glycerol (containing 0.3% impurities) because PHA accumulation on glycerol is largely determined by the degree of the purity of glycerol and the number of impurities such as methanol, salts, and esters of fatty acids, etc., which can considerably inhibit cell growth. Cultivation of *C. necator* IBP/SFU-1 on purified glycerol resulted in a cell concentration of 5.0 g/L (productivity of 1.67 g/L·d) and polymer content of 65.1% (3.26 g/L) (Figure 2). Cell biomass and polymer yield coefficients were Y_X_ = 0.38 ± 0.02 and Y_PHA_ = 0.29 ± 0.01 g/g, respectively.

*C. necator* IBP/SFU-1 cells grown on glycerol in the medium inoculated with the stock culture maintained on the nutrient agar did not show any lag phase. By contrast, three wild-type strains, *C. necator* B 5786, B 8562, and B-10646, showed 20- to 30–40-h lag phases regardless of the initial glycerol concentration in the medium. The 80-h cultivation resulted in a *C. necator* B-10646 cell concentration of 2.2 g/L, and that was higher than cell concentrations of *C. necator* B 5786 (0.7–0.8 g/L) and *C. necator* B 8562 (1.0–1.1 g/L); polymer concentrations also differed across the strains: 57, 45, and 38%, respectively. Only after the strains were adapted to glycerol by numerous re-inoculations, did the cells not show any lag phase in the early growth phases, and cell concentrations and polymer contents increased to 5.1 and 6.2 g/L and 57 and 71%, respectively [39]. Thus, production parameters of *C. necator* IBP/SFU-1 grown on glycerol were comparable to the results of cultivation of the strain *C. necator* B-10646, which was adapted to glycerol, and higher than the parameters obtained under similar conditions for other wild-type strains: *C. necator* B 5786 and B 8562, *C. necator* IPT 026, *Ps. oleovorans* NRRLB-14682, *Ps. corrugate* 388, and *Paracoccus* sp. LL1 [51,52,53].

PHA synthesis by *C. necator* IBP/SFU-1 was also investigated in experiments with fatty acids (FAs) used as the growth substrate. Metabolism of fatty acids was described as a second pathway of PHA synthesis by microorganisms in a review by Chen [16]. This pathway suggests that after β-oxidation of fatty acids, acyl-CoA is involved in the synthesis of monomers, which are then catalyzed by PHA-synthase in polymerization reactions to produce polymers. The synthesis of 3-hydroxyacyl-CoA involves 3-ketoacyl-CoA-reductases, epimerase, and, presumably, (R)-enoyl-CoA-hydratase, acyl-CoA-oxidase, and enoyl-CoA-hydratase I. This pathway is typical for the synthesis of medium-chain-length PHAs such as copolymers of 3-hydroxybutyrate and 3-hydroxyhexanoate.

Not only fatty acids but also spent cooking fats and products based on them and various plant oils, are potentially promising carbon sources for PHA synthesis. These substrates are rich in carbon and are efficiently converted to PHAs [54,55,56,57,58,59].

*C. necator* IBP/SFU-1 cell growth and PHA synthesis were studied in cultures with unsaturated (lauric, myristic, and palmitic) acids and an unsaturated, oleic, acid (Figure 2). Cultivation on oleic acid resulted in the highest cell concentration and intracellular PHA content: 8.0 g/L with a productivity of 2.67 g/L d and 78.4% (6.27 g/L). Those values were comparable to the corresponding parameters reported for *C. necator* B-10646 [60] and other strains including *Alcaligenes* sp. NCIM N5085 [61,62] grown on the same FA. Polymer concentration synthesized by *C. necator* IBP/SFU-1 was higher but cell concentration was lower than those reported for *C. necator* USMAHM13 [63].

When grown on any of the saturated FAs, *C. necator* IBP/SFU-1 produced cell concentrations comparable to those achieved on oleic acid, but polymer concentrations were lower (Figure 2): 65.9 and 64.2% on lauric and myristic acids, respectively, and only 49% on palmitic acid. Similarly lower parameters were obtained on saturated FAs compared to oleic acid in studies of *C. necator* USMAHM13 [63] and *C. necator* USMAA2-4 [62]. The most likely explanation lies in the physiological and biochemical properties of each strain and its ability to metabolize a certain fatty acid. For the strain *C. necator* USMAA2-4, FAs, as effective substrates for bacterial cells, were ranked as follows: oleic acid > palmitic acid > myristic acid > lauric acid [62]. For *C. necator* USMAHM13, by contrast, cells grown on palmitic acid showed the lowest biomass concentration and PHA content [63].

Three plant oils, palm oil, Siberian oilseed oil, and sunflower seed oil, were tested as carbon sources for *C. necator* IBP/SFU-1; the oils differed considerably in their FA compositions as shown in a study by Volova et al. (2020) [64]. Sunflower seed oil was a liquid oil with the highest contents of linoleic acid (18:2ω6) and oleic acid (18:1ω9); other acids (20:0, 22:0, and 24:0) were present in small amounts (0.4–1.1%); the saturation index of the oil was 0.14. Siberian oilseed oil contained 15 FAs, and unsaturated FAs were prevalent as well (constituting about 90%); its saturation index was similar to that of refined sunflower seed oil (0.11). Palm oil was a semisolid or solid plant oil; the saturation index of the tested sample was 0.97, suggesting the prevalence of saturated FAs. Thus, the tested oils differed considerably in their FA compositions.

Cultivation of *C. necator* IBP/SFU-1 on palm oil resulted in the highest production parameters (Figure 2). Cell concentration was 8.2 g/L (with the highest productivity of 2.73 g/L · d) and intracellular polymer content was 80% (6.56 g/L). That was comparable with the results obtained in the 72-h culture of the wild-type strain *C. necator* B-10646-7.1 g/L and 7–82.7%, respectively [64], and superior to the production parameters of another wild-type strain, *C. necator* H16-5.6 g/L and 77%, respectively [65]. Not all *C. necator* strains, however, are able to utilize palm oil, the wild-type strain *C. necator* USMAA2-4 failed to grow on palm oil [62].

Results of *C. necator* IBP/SFU-1 cultivation on sunflower seed oil were lower compared to palm oil and were similar to the results obtained for *C. necator* B-10646: a cell concentration of 4.3 g/L, a productivity of only 1.77 g/L d, and a polymer content of 38.7% [64]. Lee et al. (2008) reported a similar cell concentration but higher polymer content (72%) for *C. necator* H16 [65]. In another study [66], however, cell concentration produced by the same strain, H-16, was higher (9.4 g/L) but polymer content lower (49%).

Siberian oilseed oil was first tested as a source for PHA synthesis in a culture of C. necator B-10646 [64]; the resulting cell concentration was 5.8 g/L and polymer content 59%. In the present study, C. necator IBP/SFU-1 produced similar results. All fat-containing sources were suitable substrates for the growth of the new strain and for PHA synthesis, although production parameters of the cultures differed somewhat. The yield coefficients for palm oil, Siberian oilseed oil, and fatty acids were 0.76 ± 0.01 g biomass/g substrate and 0.48 ± 0.02 g polymer/g substrate, and those values were 1.5–2.0 times higher than the yield coefficients obtained for sunflower seed oil, sugars, and glycerol.

Our results showed that the newly isolated wild-type strain *Cupriavidus necator* IBP/SFU-1 is able to grow and synthesize PHAs not only on the highly specialized fructose but also on the more available glucose, fatty acids, various plant oils, and glycerol as well as under autotrophic growth conditions, on CO_2_ mixed with hydrogen and oxygen. Moreover, the replacement of the sugar sources by any of the other carbon sources tested in the present study did not lead to any long adaptation or lag-phase. This is a definite advantage of the new strain over the other known *Cupriavidus* representatives, some of which are unable to assimilate glucose or palm oil, show low productivity on glycerol, and need a long time to adapt to other carbon sources after being reinoculated from sugar-containing media.

### 3.3. Properties of PHAs Synthesized by Cupriavidus Necator IBP/SFU-1 from Various Carbon Sources

Properties of PHAs are determined by their structure, primarily, the structure of the side chains in the polymer carbon chain and the distance between the ester groups in a molecule [10,11,14,16]. The ability of the PHA producing strains to synthesize polymers with different compositions is determined by their biochemical properties, organization of the intracellular enzyme system of PHA synthesis, and substrate specificity of PHA-synthase-the enzyme catalyzing monomer polymerization reactions. The factor of the culture medium that influences the chemical composition of the PHAs is the carbon source.

It was previously believed that *Cupriavidus* species were only able to synthesize short-chain length PHAs: P(3HB) and P(3HB-co-3HV). *Cupriavidus* strains had been considered as producers of short-chain-length PHAs (scl-PHAs) until some of the strains of this genus were found to be able to synthesize PHAs containing medium-chain-length monomers (3-hydroxyhexanoate, 3-hydroxyoctanoate) when cultivated in the medium supplemented with the corresponding precursor substrates [28,29]. The ability to synthesize a wide range of PHAs including short- and medium-chain-length monomers makes these strains particularly promising PHA producers.

**Figure 3:**
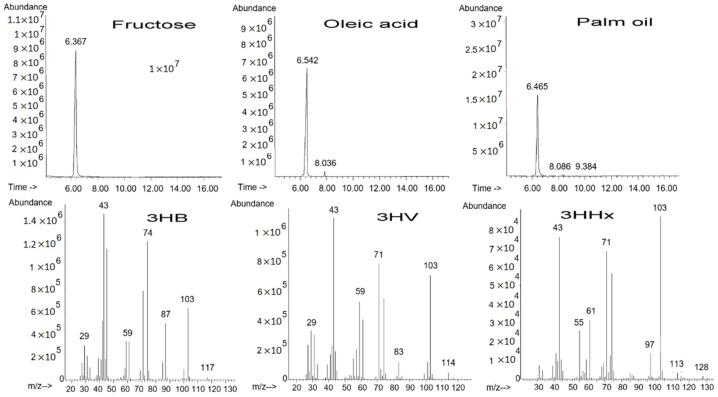
Ion chromatograms with mass spectra of methyl esters of the monomers constituting PHAs synthesized by *Cupriavidus necator* IBP/SFU-1 from different carbon sources with retention times: methyl-3-hydroxybutyrate (3HB)—6.367–6.542; methyl-3-hydroxyvalerate (3HV)—8.036–8.086; methyl-3-hydroxyhexanoate—9.384 min. 1.1 × 10.

Cultivation of *C. necator* IBP/SFU-1 on various carbon sources resulted in differences in not only production parameters and substrate utilization efficiency but also the composition and properties of the synthesized polymers. Ion chromatograms and mass spectra of the polymers synthesized by *C. necator* IBP/SFU-1 from various carbon sources provide evidence for the effect of the type of the carbon source on PHA composition (Figure 3). Cultivation of the strain on oleic acid and all plant oils resulted in the synthesis of PHA copolymers. The P(3HB-co-3HV) copolymers containing 3-hydroxyvalerate (3HV) monomers as the minor component of the carbon chain of the dominant 3-hydroxybutyrate (3HB) were synthesized from oleic acid. The P(3HB-co-3HV-co-3HHx) terpolymers composed of 3HB (above 99%) and minor fractions of 3-hydroxyvalerate (3HV) and medium-chain-length 3-hydroxyhexanoate (3HHx) were synthesized from three plant oils (Table 1). Those results were consistent with the data reported in another study [64]. In that study, the wild-type strain *C. necator* B-10646, which when grown on sugars synthesized the P(3HB) homopolymer, was grown on plant oils (palm oil, Siberian oilseed oil, and sunflower seed oil) and produced a PHA terpolymer composed of the dominant 3HB (97–99 mol.%) and the minor fractions of 3-hydroxyvalerate monomers (0.9–1.9 mol.%) and the medium-chain-length 3-hydroxyhexanoate (0.4–1.3 mol.%).

**Figure 4:**
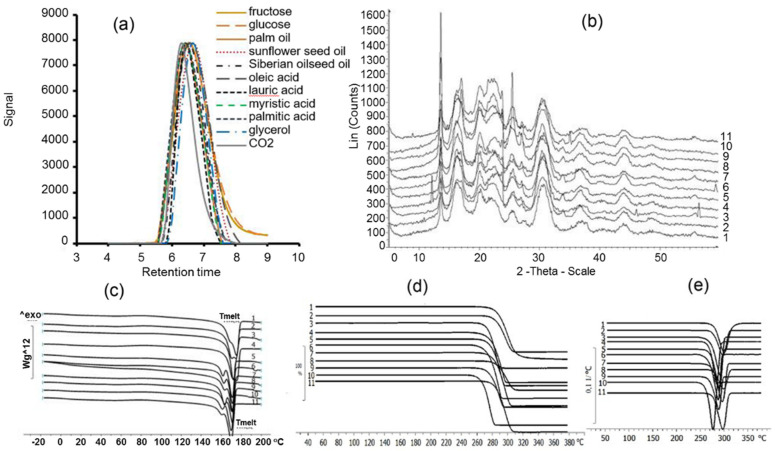
Physicochemical properties of PHAs synthesized by *Cupriavidus necator* IBP/SFU-1 from various carbon sources: (**a**) molecular weight distribution chromatogram, (**b**) the X-ray, (**c**) DSC, (**d**) TGA, (**e**) DTG (numbering of Samples 1–11 is the same as in Table 1).

The main differences in the physicochemical properties of the polymers were between their molecular-weight properties—a major characteristic of high-molecular-weight compounds, affecting polymer processing into products and their mechanical qualities (Figure 4, Table 1). P(3HB) synthesized under autotrophic growth conditions on CO_2_ had the highest molecular weight (M_n_ and M_w_) and decreased polydispersity (Ð). The other P(3HB) specimens, which were synthesized from organic sources, showed reduced molecular weight and increased polydispersity. These results are consistent with the data on other wild-type strains of hydrogen-oxidizing bacteria (*C. necator* B 5786, *C. necator* B 8562, *C. necator* B-10646), although P(3HB) synthesized on CO_2_ by those strains had much higher absolute values of weight-average molecular weight, reaching 1000 kDa and even more [46]. The present study showed a decrease of 200 and even 350 kDa in the M_w_ of P(3HB) synthesized by *C. necator* IBP/SFU-1 from fatty acids and plant oils and an increase in Ð to 3.2–4.5 compared to the results obtained on CO_2_. A previous study demonstrated [64], that although polydispersity of the PHAs synthesized by *C. necator* B-10646 grown on plant oils increased considerably (to 4–5), their M_w_ values ranged between 670 and 780 kDa, and the PHAs synthesized from palm oil had the lowest molecular weight. It is likely that the decrease in molecular weight of PHAs synthesized by bacterial cells grown on oils was caused by the metabolism of triacylglycerols in the culture medium containing oil and the release of glycerol, which functioned as a chain transfer agent during PHA polymerization [67], resulting in a lower-molecular-weight polymer. The most considerable reduction in the molecular weight of the P(3HB) specimens synthesized by *C. necator* IBP/SFU-1 from glycerol supports this suggestion.

A number of studies analyzed the properties of PHAs synthesized from glycerol, placing a special emphasis on molecular-weight properties because glycerol has been reported to function as a chain transfer (CT) reaction agent in PHA polymerization, resulting in the formation of low-molecular-weight PHA. In case of a CT reaction, the PHA chain number increases in inverse proportion to the PHA molecular weight [68]. However, some of the data on the M_w_ of the polymers synthesized on glycerol are contradictory. There are studies reporting the reduction in the M_w_ of P(3HB) synthesized from glycerol [52,69], but other authors show rather high M_w_ values of the polymer produced from glycerol, which reach 620 and 750 kDa [70] and even 790–960 kDa [71]. The study of the properties of P(3HB) synthesized by *C. necator* B-10646 from glycerol demonstrated that the degree of purity of glycerol affected polymer molecular weight [39]. The polymer synthesized from purified and distilled glycerol had reduced M_w_-355 and 420 kDa, but the M_w_ of the polymer synthesized from crude glycerol was even lower, 304 kDa.

PHAs are semi-crystalline polymers, as crystallization does not occur in all regions of the material. The degree of crystallinity is the least studied aspect of PHAs. P(3HB) has been generally recognized as a high-crystallinity polymer, in which the crystalline phase prevails over the amorphous phase, constituting from 65–67 to 80% [72].

The division of the polymer into crystallites and amorphous regions and determination of the degree of crystallinity are a first-approximation structural scheme, which describes the supramolecular structure. As the new data are obtained, this scheme is developed and improved. However, the data on the degree of crystallinity are widely used in scientific and technical research because this parameter directly determines the mechanical and physical properties of the polymer. The degree of crystallinity determines hardness, density, transparency, and diffusion. The higher the degree of crystallinity, the harder and stronger, but more brittle the product is.

In the present study, differences in the degrees of crystallinity of the PHAs synthesized from the carbon sources were not statistically significant. The C_x_ values varied within a rather narrow range (65–72%) regardless of the type of carbon source and the structure of the polymers (Table 1).

The thermal properties of PHAs and their ability to crystallize in a native state are important parameters that determine the thermomechanical properties and melt processability of the polymer. The considerable difference between the melting point and the thermal degradation temperature is an important processing property of the polymer and owing to this property, the polymer can be processed into different products from melts, using generally accepted methods (solvent molding, extrusion, injection molding). P(3HB) has a softening point at approximately 110 °C and crystallization temperature (T_c_) between 65 and 91 °C. No significant differences were revealed between the thermal properties of the PHAs synthesized from various carbon sources by the new strain *C. necator* IBP/SFU-1 (Table 1, Figure 4c–e). All PHA specimens had similar melting points (T_melt_) and thermal degradation temperatures (T_degr_), with no statistically significant differences between them. T_melt_ values ranged between 160 and 172 °C and T_degr_ values between 267 and 285 °C. Previous studies showed that glycerol [39] and plant oils [64] did not affect the thermal properties of PHAs synthesized by *C. necator* B10646.

The present study demonstrated that PHA composition and properties were determined not only by the nutrient sources such as precursor substrates supplementing the main carbon source but also by the characteristics of the PHA producing strain and the type of the growth carbon source. *C. necator* IBP/SFU-1 was found to be capable of synthesizing short- and medium-chain-length PHAs even when cultivated in the medium that had not been supplemented with precursor substrates. Hence, the addition of precursors of different monomers to the culture medium can be expected to increase the fractions of 3HV, 3HHx, and, probably, other monomers in the PHA copolymers synthesized by this strain.

The review of the literature on physicochemical properties of PHAs revealed that the data differed substantially even when the specimens described in the studies were similar in their chemical composition. The authors of the study [73] noted that the data on the molecular weight of P(3HB) reported by different researchers differed by one order of magnitude and the data on P(3HB) T_melt_ varied between 162 and 197 °C. The T_melt_ of P(3HB-co-3HV) copolymers with the same 3HV content ranged between 56 and 186 °C [10]. In the study [73], 3HV content of up to 20–22 mol.% did not influence the degree of crystallinity of P(3HB-co-3HV), whereas, in another study [74], the C_x_ of the same copolymer was considerably reduced (to 5–9%). The degree of crystallinity of the P(3HB-co-3HHx) copolymer containing 3HHx approximately 12–18 mol.% was 38–40% [74], but in the study [75], the same degree of crystallinity was determined for the copolymer with 3HHx content of only 1.5 mol.%. The reason for such differences could be that the authors used different strains and carbon sources, collected polymer samples in different phases of the culture, used dissimilar methods of recovery and purification of polymers, etc. Thus, these factors should be taken into account in studies of the properties of PHAs and PHA-based products.

In addition, there are no definitive data showing how the basic properties of PHAs change depending on the composition of the medium and cell growth conditions and how these changes affect the properties of the PHA products. At the same time, some researchers reported that the chemical composition of PHA copolymers and the monomers constituting them influenced the surface microstructure and properties of polymer films [42,76] and their biological compatibility and blood cell response [77]. Replacement of glucose by plant oils containing glycerol not only resulted in changes of molecular weight and crystallinity of P(3HB) synthesized by *C. necator* H16 but also considerably affected the structure and properties of nonwoven membranes fabricated using electrospinning technique [78]. A similar effect of the type of carbon source on the properties of polymer films was described in [76]: comparison of the surface properties of the films prepared from P(3HB) synthesized by *C. necator* B-10646 from glucose and glycerol revealed a considerable reduction in water contact angles of the surfaces of the films prepared from the polymer synthesized from glycerol.

**Figure 5:**
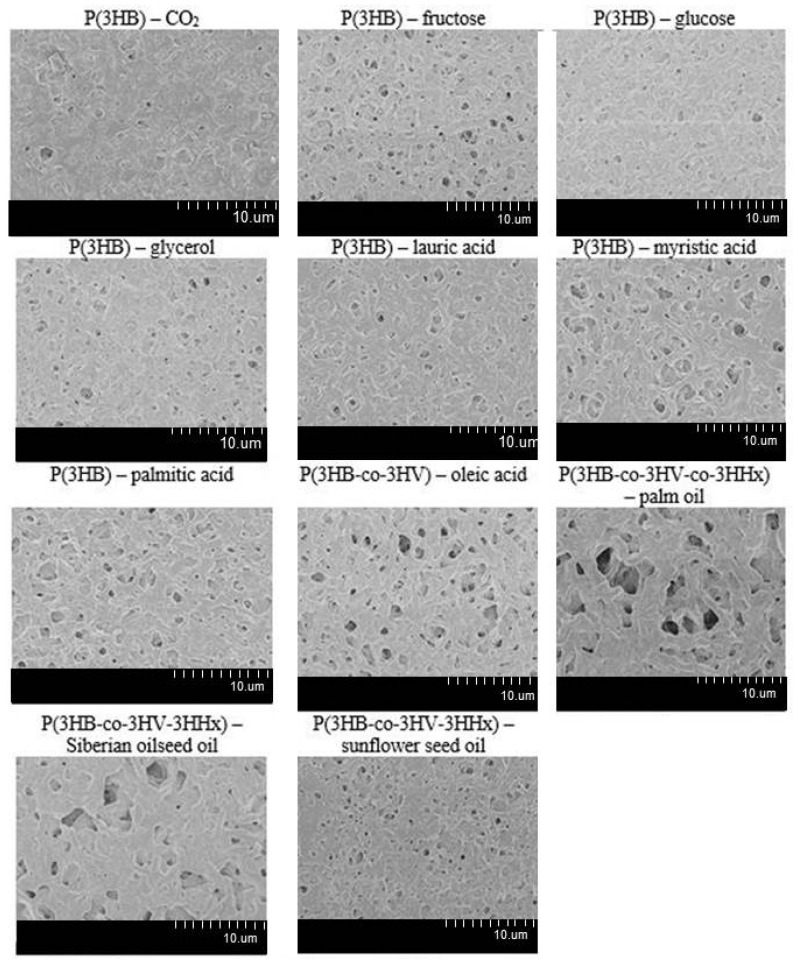
SEM images of the films fabricated from PHA synthesized from different carbon sources by *Cupriavidus necator* IBP/SFU-1.

**Figure 6:**
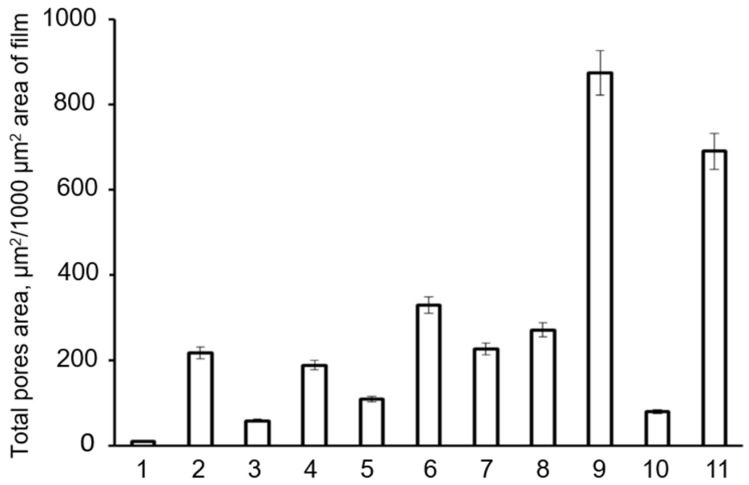
Total pores areas of films fabricated from PHAs synthesized from different carbon sources by *Cupriavidus necator* IBP/SFU-1 (numbering of Samples 1–11 is the same as in Table 1).

**Figure 7:**
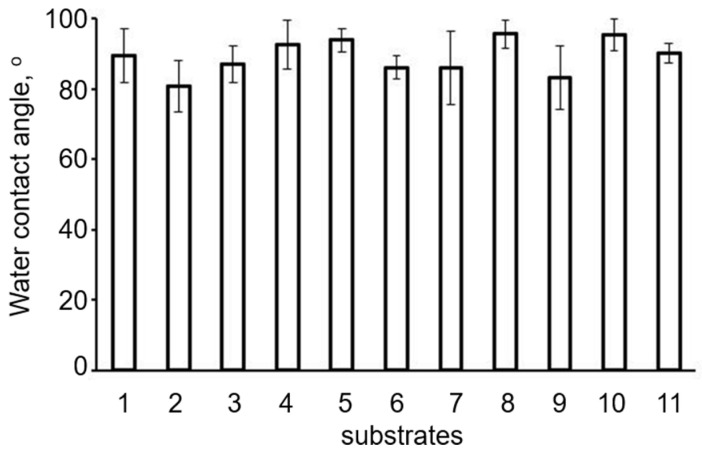
Water contact angles of the films prepared from PHAs synthesized from various carbon sources by *Cupriavidus necator* IBP/SFU-1 (numbering of Samples 1–11 is the same as in Table 1).

Figure 5, Figure 6 and Figure 7 show results of examining the films fabricated from PHAs synthesized from various carbon sources by *C. necator* IBP/SFU-1. SEM images illustrate differences in surface morphology (Figure 5).

The surfaces of the films fabricated from PHAs synthesized from various carbon substrates differed in the number and size of pores. That was most likely caused by differences in the kinetics of crystallization during the formation of the films, as they dry and the solvent evaporates.

Comparison of the porosity of films fabricated from one type of PHA, poly(3-hydroxybutyrate), showed that the number of pores was similar for all seven samples (32.8–46.5/1000 μm^2^) while the pore sizes of these samples differed significantly. Thus, the average pore area was minimal, 1.9 and 2.4 μm^2^, for the films fabricated from P(3HB) synthesized from glucose and lauric acid, respectively. All copolymer films, except the films fabricated from the P(3HB-co-3HV-co3HHx) terpolymer synthesized from sunflower oil, had larger pore areas. The average pore area was 14.0, 21.7, and 24.7 μm^2^, respectively, for films fabricated from the P(3HB/3HV) copolymer synthesized from oleic acid and films fabricated from P(3HB-3HV-co-3HHx) synthesized from palm oil and Siberian oilseed oil (data not shown).

These differences are reflected in the integrated indicator, the total pore area (Figure 6).

The lowest values of the total pore area (11 µm^2^/1000 µm^2^ of the film surface area) were found for the films fabricated from P(3HB) synthesized under autotrophic conditions. The total pore areas of 59 and 110 μm^2^/1000 μm^2^ were found for the films of P(3HB) synthesized from glucose and lauric acid and for the film synthesized from sunflower oil (80 μm^2^/1000 μm^2^). The pore areas of the copolymer films were several times greater. The highest values (690 and 874 µm^2^/1000 µm^2^) were found for the films fabricated from P(3HB-co-3HV-3HHx) synthesized from Siberian oilseed oil and palm oil. Thus, films with different porosities can be fabricated using PHAs synthesized from various C-substrates differing in their monomer compositions.

Thus, solution-cast films prepared from PHAs with different chemical compositions had surfaces differing in the number and size of pores. That could be associated with differences in the crystallization kinetics during solvent evaporation and formation of films from polymers with different degrees of crystallinity, which could affect the attachment and development of eukaryotic cells. The carbon source used to synthesize a PHA did not affect the water contact angle, which is an indirect indicator of surface hydrophilicity. The water contact angles of all films were similar (Figure 6). Films fabricated from P(3HB) specimens synthesized from glucose, glycerol, and FAs showed almost equal water contact angles.

The decrease in the values of the water contact angle of the films (Figure 7) prepared from P(3HB) synthesized from fructose and P(3HB-co-3HV-co-3HHx) synthesized from palm oil to 80.8 ± 7.33 and 83.3 ± 9.0°, respectively, was non-significant (at the confidence level α = 0.05). Another study [76], though, demonstrated a significant decrease in the water contact angles of the films prepared from P(3HB) specimens synthesized from glycerol compared to sugars. In that study, however, polymer synthesized from glycerol showed a considerably (50–55%) decreased crystallinity. The increase in the values of the water contact angle of the films prepared from PHA copolymers synthesized from sunflower seed oil and oleic acid (95.5–95.7°) was not statistically significant either. Thus, none of the film types prepared from PHAs synthesized from various carbon sources by *C. necator* IBP/SFU-1 and having similar degrees of crystallinity and thermal properties showed any significant differences in the values of the water contact angle.

## 4. Conclusions

The bacterial strain isolated from the soil in the Krasnoyarsk Territory was studied as a PHA producer. The bacterial strain was identified as *Cupriavidus necator* IBP/SFU-1 based on the morphological, culture, and molecular-genetic characters. The strain was used as inoculum in suspension culture and batch-cultured to study cell growth and PHA synthesis from different carbon sources. The strain was found to be able to grow and synthesize PHAs under autotrophic conditions on CO_2_ mixed with H_2_ and O_2_ and showed a broad organotrophic potential towards different carbon sources: sugars, glycerol, fatty acids, and various plant oils. The highest cell concentrations and PHA content were produced in the cultures with palm oil and oleic acid used as carbon sources. Cultivation on the widely available palm oil resulted in cell concentration and intracellular polymer content reaching 8.2 and 6.56 g/L, respectively, and the highest productivity (2.73 g/L d) and high (80%) polymer content. On oleic acid, cell concentration, and intracellular polymer content were 8.0 and 6.27 g/L, corresponding to biomass productivity of 2.67 g/L · d and polymer content of 78.4%. Results obtained on sugars (fructose and glucose) did not differ considerably, cell concentrations were 7.0 and 7.7 g/L and intracellular polymer contents were 5.68 and 6.33 g/L, respectively, with productivities of 2.33 and 2.57 g/L d and polymer content of 81.1 and 84.3%. Cultivation on purified glycerol produced somewhat lower results, cell concentration and intracellular polymer content were 5.0 and 3.26 g/L and productivity reached 1.67 g/L d and 65.1%. Production parameters of the strain grown on other substrates (saturated fatty acids and sunflower seed oil and Siberian oilseed oil) were even lower.

The study showed that PHA chemical composition and properties were determined by the carbon source used. When grown on oleic acid, the strain synthesized the P(3HB-co-3HV) copolymer; on Siberian oilseed oil, sunflower seed oil, and palm oil—the P(3HB-co-3HV-co-3HHx) terpolymer, and on the other substrates—the P(3HB) homopolymer. In all copolymer types, 3-hydroxybutyrate constituted over 99 mol.%, and 3-hydroxyvalerate and the medium-chain-length 3-hydroxyhexanoate were present in minor amounts. The type of the carbon source mainly influenced molecular-weight properties of PHAs: molecular weight and polydispersity. P(3HB) synthesized under autotrophic growth conditions, from CO_2_, had the highest number-average (290 ± 15 kDa) and weight-average (850 ± 25 kDa) molecular weights and the lowest polydispersity (2.9 ± 0.2). All polymers synthesized from organic carbon sources, regardless of PHA composition, showed increased polydispersity and reduced molecular weight; P(3HB) synthesized from glycerol had the lowest M_n_ and M_w_. All PHAs, regardless of the type of the carbon source used, had similar degrees of crystallinity, with the ordered phase prevailing in the polymers (64–72%); no effect of the carbon source on thermal properties of the PHAs was found either. The type of the carbon source determined not only PHA composition and properties but also the surface microstructure and porosity of polymer films. Results obtained in this study show that the strain *Cupriavidus necator* IBP/SFU-1 has a broad organotrophic potential and is capable of synthesizing PHAs from various carbon substrates, producing high cell concentrations and intracellular contents of polymers, including short- and medium-chain-length copolymers. Thus, this strain can be regarded as a promising P(3HB) producer from palm oil, oleic acid, and sugars (fructose and glucose) and as a producer of P(3HB-co-3HV) from oleic acid and P(3HB-co-3HV-co-3HHx) from palm oil.

## Figures and Tables

**Table 1:** Composition and properties of PHAs synthesized by *Cupriavidus necator* IBP/SFU-1 from various carbon sources.

No.	Carbon Source	Monomer Composition of PHA, mol.%			M_n_, kDa	M_w_, kDa	Ð	C_x,_ %	T_melt_, °C	T_degr_, °C
		3HB	3HV	3HHx						
1	CO_2_	100.0	-	-	290 ± 15	850 ± 25	2.9 ± 0.2	67	174.2	280.1
2	Fructose	100.0	-	-	136 ± 9	499 ± 20	3.7 ± 0.4	65	172.4	284.7
3	Glucose	100.0	-	-	137 ± 1	557 ± 24	4.1 ± 0.2	64	171.6	279.9
4	Glycerol	100.0	-	-	108 ± 4	409 ± 11	3.9 ± 0.3	69	172.8	274.9
5	Lauric acid	100.0	-	-	162 ± 11	510 ± 10	3.2 ± 0.2	63	161.0 170.6	279.0
6	Myristic acid	100.0	-	-	144 ± 10	648 ± 12	4.5 ± 0.3	65	162.1 170.8	275.8
7	Palmitic acid	100.0	-	-	174 ± 8	673 ± 4	3.9 ± 0.2	72	162.9 171.2	283.4
8	Oleic acid	99.70	0.3	-	118 ± 4	526 ± 2	4.5 ± 0.2	66	162.7 170.3	285.9
9	Palm oil	99.80	0.07	0.13	165 ± 6	682 ± 9	4.1 ± 0.2	66	162.5 171.1	277.6
10	Sunflower seed oil	99.77	0.21	0.02	124 ± 2	479 ± 4	3.9 ± 0.1	70	160.7 169.6	267.6
11	Siberian oilseed oil	99.71	0.25	0.04	149 ± 9	511 ± 21	3.4 ± 0.2	65	160.5 169.8	285.3

“-” no such monomer in the PHA.

## Data Availability

Not applicable.
